# Supplementary material for: GC–MS Untargeted Analysis of Volatile Compounds in Four Red Grape Varieties (Vitis vinifera L. cv) at Different Maturity Stages near Harvest
Source: Foods. 2022 Sep 11;11(18):2804. doi: 10.3390/foods11182804 (PMC9497989; doi:10.3390/foods11182804)
Supplement: Supplementary file 1 [file foods-11-02804-s001.zip › Supplementary information1.pdf]

Supplementary information

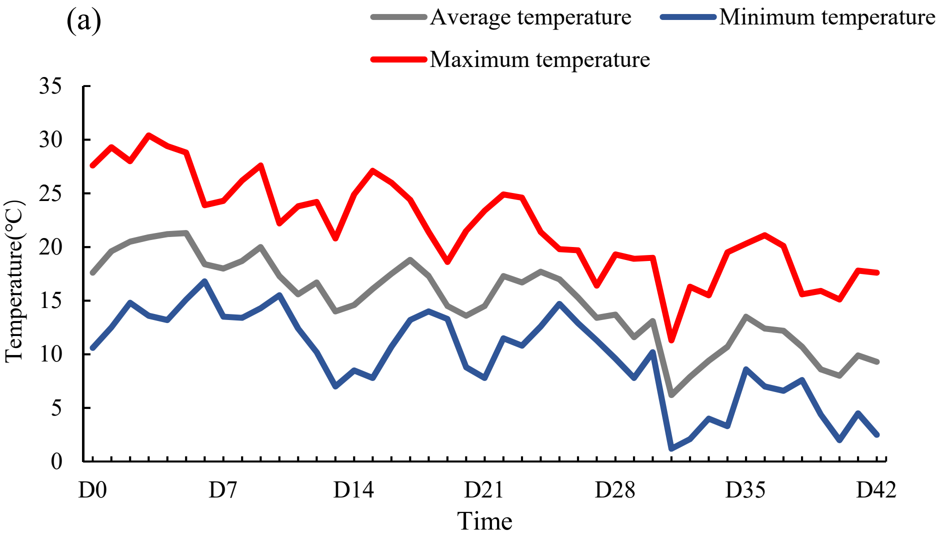

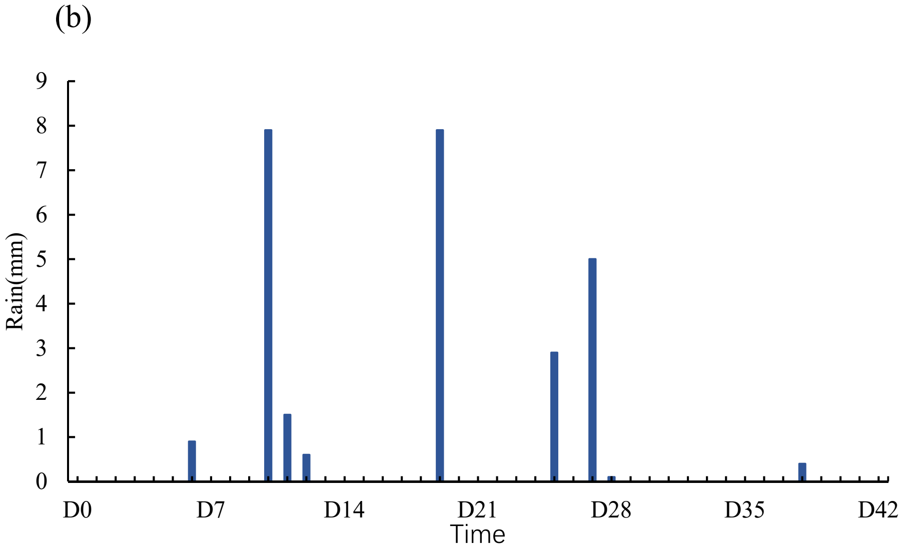

Figure S1. The temperature (a) and rainfall (b) of the vineyard during the ripening process of the grape.

Note: Average temperature, maximum temperature, minimum temperature and rainfall during the ripening process of grape were supplied by China Meteorological Data Sharing Service System (http://data.cma.cn).

Table S1

Standard curves and related parameters of IBMP in juice samples.

Compound	Linear Range (ng/L）	Equation	R^2	LOD (ng/L)	LOQ (ng/L)	Recovery [a] (%)
IBMP	0-50	y = 8049.1x + 11880	0.9956	0.34	1.03	94.13-109.87

Note: a. The recovery was determined in the juice sample with IBMP 20 ng/L and 35 ng/L mixed standards.
